# Identification of embryonic yield-related serum biomarkers using proteomic analysis in ovine superovulation

**DOI:** 10.3389/fvets.2025.1675287

**Published:** 2025-11-04

**Authors:** Xiaohu Su, Yijing Zhu, Zirong Guo, Shuang Qiu, Quan Liu, Chao Bian, Liqiang Chen, Hideaki Yamashiro, Guangnan Liu, Zhanwen Zhang, Liguo Zhang, Bin Tong

**Affiliations:** ^1^School of Life Sciences, Inner Mongolia University, Hohhot, China; ^2^Ulanqab Animal Husbandry Workstation, Ulanqab Agriculture and Animal Husbandry Bureau, Ulanqab, China; ^3^Department of Tumor Radiotherapy, Inner Mongolia People’s Hospital, Hohhot, China; ^4^Department of Agriculture, Faculty of Agriculture, Niigata University, Niigata-shi, Niigata-ken, Japan; ^5^Animal Husbandry and Veterinary Services Center, Chahar West Middle County Agriculture and Animal Husbandry Bureau, Ulanqab, China

**Keywords:** superovulation, serum proteome, biomarker, sheep, donor selection

## Abstract

Superovulation is an efficient technology for the propagation of excellent livestock. Blood proteins other than reproductive hormones might be related to the outcome of superovulation. In this study, to identify potential protein biomarkers, the serum proteome of superovulation donor ewes was analyzed. The ewes were classified into spontaneous estrus (SE) and induced estrus (IE) groups, and their blood samples were collected before the first follicle-stimulating hormone (FSH) injection. Then, high (HEY) and low embryonic yield (LEY) populations of each group were identified based on the total embryonic number. Five donors from each population were selected for serum proteomic analysis. Finally, partially differentially expressed proteins (DEPs) were verified using the enzyme-linked immunosorbent assay (ELISA). Averages of 18.6 ± 12.4 and 14.4 ± 8.7 total embryos were collected from the SE and IE groups, respectively. We identified 13 and 12 DEPs in the SE and IE groups, respectively, after the comparison of the HEY and LEY populations of both groups. Furthermore, the ELISA revealed that three DEPs—ACAA1, B2MG, and ADAMTS13—in the SE group and two DEPs—B2MG and APO-F—in the IE group exhibited a significant linear correlation with the total number of embryos. This study showed that ACAA1, B2MG, ADAMTS13, and APO-F could be used as potential biomarkers for donor selection in ovine superovulation. Our results provide novel insights into the relationship between blood proteins and ovarian function and offer a theoretical basis for the prediction of superovulation outcomes.

## Introduction

In livestock breeding, the propagation of excellent individuals is necessary. The reproductive rate of sheep is relatively low. Therefore, the application of superovulation technology has been shown to be useful for the rapid propagation of high-quality sheep. However, the outcome of superovulation is inconsistent, which may be attributable to multiple factors, including breed, administration protocols, age, hormone source and purity, nutritional status, and reproductive status (1). Despite implementing strict screening conditions for donors and maintaining consistent processes, individual outcomes can vary widely. Therefore, the exploration of better strategies for donor selection or outcome prediction is particularly necessary to improve the efficiency of superovulation.

In essence, superovulation refers to stimulating the maturation of original small follicles in the ovaries. Therefore, the detection of the status of original small follicles via ultrasound is an efficient way to predict the embryonic yield (2–7). Nevertheless, this process can be complex and necessitates technical know-how. In addition, the stress endured by donors cannot be overlooked. Therefore, the use of non-invasive methods would be beneficial in this context. Previously, we found that follicle-stimulating hormone (FSH), progesterone (P4), anti-müllerian hormone (AMH), phosphatidyl choline (PC)-related metabolites, and some amino acids in the blood were associated with superovulation results (8). Studies have shown that blood proteins are associated with reproductive capacity. For instance, plasma protein Z concentration has been demonstrated to be associated with adverse pregnancy outcomes, including recurrent miscarriage, stillbirth, preeclampsia, intrauterine growth restriction, and placental abruption (9). The human leukocyte antigen in blood has been shown to be associated with recurrent implantation failure (10). However, the literature on the relationship between blood proteins and the effectiveness of superovulation in livestock is limited.

The East Friesian sheep is a type of German dairy sheep breed that is well-known for its high milk yield (11). In this study, the East Friesian ewes with similar status during the breeding season were selected as superovulation donors. Their blood samples were collected before the FSH injection. Serum proteomic analysis was performed using the tandem mass tag (TMT)-based liquid chromatography–tandem mass spectrometry (LC–MS/MS) technique. Furthermore, the expression levels of partially differentially expressed proteins (DEPs) were detected using the enzyme-linked immunosorbent assay (ELISA). Our findings might help screen novel biomarkers and develop relevant theories to improve the outcome of ovine superovulation.

## Methods

### Ethics statement and consent to participate

This study was approved by the Inner Mongolia University Research Ethics Committee (2021002). All experiments were performed according to Chinese laws and institutional guidelines. The study protocol was reported in accordance with the ARRIVE guidelines.^1^ Informed consent was obtained from all owners for the use of their sheep.

### Superovulation donors’ feeding location and diet composition

A total of 103 healthy East Friesian ewes, aged between 2 and 2.5 years and with a body weight ranging from 60 to 63 kg, were selected as donors for superovulation. The donors were fed at the Fengdongzhiying Husbandry Technology Co., Ltd. in Ulanqab (Ulanqab, Inner Mongolia, China) using a barn feeding system (43°28′ north latitude and 114°49′ east longitude, altitude 2,150 m above sea level).

The donors were fed a total mixed ration comprising 14% alfalfa hay, 27% *Leymus chinensis*, 4% corn grass, 15% whole corn silage, 15% corn grain, and 25% ewe concentrate supplement, with a forage: concentrate ratio of 72:28.

### Superovulation and embryo collection protocols

Ovine superovulation and embryo collection were conducted, as described previously (8), in the breeding season of November. At the beginning of the study, the estrus status of the donors was detected using rams. The ewes that accepted stable presentation were classified into the SE group (*n* = 32), and the others were classified into the IE group (*n* = 71). The SE group was treated as follows: The estrus day was designated as D0. From D13 to D15, each donor received a total of 250 μg of FSH (Stimufol^®^, Belgium), administered in six progressively decreasing doses. For the first and last FSH injections, 150 μg of PG (GINTENBIOTECHNOLOGY, Beijing, China) was injected synchronously. Then, the estrus donors were artificially inseminated with 100 μL of fresh semen; 100 IU of LH (GINTENBIOTECHNOLOGY, Beijing, China) was injected synchronously. On D22, embryos were collected via surgical uterine flushing after the ewes were anesthetized with 5 mL of lidocaine hydrochloride (0.02 g/mL, CSPC, Hebei, China).

The IE group was treated as follows: The day of Controlled Internal Drug Release (CIDR) (Zoetis, New Zealand) insertion was designated as D0. From D10 to D12, each donor received a total of 250 μg of FSH, administered in six decreasing doses. During the first FSH injection, 250 IU of PMSG (GINTENBIOTECHNOLOGY, Beijing, China) was injected synchronously; during the fifth FSH injection, 150 μg of PG was injected synchronously. The CIDR was removed at the time of the final FSH injection. Then, the estrus donors were artificially inseminated with 100 μL of fresh semen; 100 IU of LH was injected synchronously. On D19, embryos were collected using the same method adopted for the SE group.

### Blood sampling

Blood samples from the SE and IE groups were collected in the morning on D13 and D10, respectively, that is, the time before the first FSH injection. Blood samples were collected in vacuum blood collection tubes. Serum was obtained by centrifugation at 10,000 × g for 10 min. All serum samples were stored in liquid nitrogen until further use.

### Serum proteomic analysis

The HEY and LEY populations were divided based on the total embryonic number. The donors were arranged in descending order based on the total embryonic number, and the first and last one-third populations were designated as HEY and LEY populations, respectively. Five donors from each population were selected for serum quantitative proteomic analysis.

First, 40 μL of each serum sample was diluted tenfold with binding buffer and passed through a resin column to remove albumin. The vacuum freeze-dried samples were redissolved in SDS lysate to obtain a 1 mM solution. The protein concentration was determined following the instructions provided in the BCA kit. The integrity of the results was validated using SDS-PAGE electrophoresis and Coomassie Brilliant Blue staining. Then, 50 μg of protein from each sample was digested with trypsin. The resulting peptides were labeled with the 10-plex TMT reagent, in accordance with the manufacturer’s instructions (Thermo Scientific, Wilmington, DE, USA).

All analyses were performed using a Q-Exactive mass spectrometer (Thermo Scientific, Wilmington, DE, USA) equipped with a Nanospray Flex source (Thermo Scientific, Wilmington, DE, USA). The samples were loaded onto and separated by a C18 column (15 cm × 75 μm) on an EASY-nLC™ 1,200 system (Thermo, USA). The flow rate was 300 nL/min, and the linear gradient was as follows: 0 ~ 40 min, 5–30% B; 40 ~ 54 min, 30–50% B; 54 ~ 55 min, 50–100% B; and 55 ~ 60 min, 100% B. Mobile phase A consisted of 0.1% FA in water, and mobile phase B consisted of 80% ACN/0.1% FA in water. Full MS scans were acquired in the mass range of 300–1,600 m/z, with a mass resolution of 70,000 and an AGC target value of 1e6. The 10 most intense peaks in the MS scan were fragmented using higher-energy collisional dissociation (HCD) with an NCE of 32. MS/MS spectra were obtained at a resolution of 17,500, with an AGC target of 2e5 and a maximum injection time of 80 ms. The Q-E dynamic exclusion was adjusted to 30.0 s and run under positive mode.

All of the Q Exactive raw data were searched thoroughly against the sample protein database of Proteome Discoverer (version 2.4). The database search was performed with trypsin digestion specificity. The alkylation of cysteine was considered a fixed modification during the database search. The labeling method was selected for protein quantification. A global false discovery rate (FDR) of 0.01 was applied, and protein groups considered for quantification were required to have at least two peptides.

The functional analysis of DEPs was performed using GO annotation, KEGG enrichment analysis, and PPI network analysis. GO annotation was performed via the website http://www.geneontology.org/. KEGG was performed via the website http://www.geneontology.org. PPI network analysis was performed using the Cytoscape Plug-in Network Analyzer.

### Elisa

A total of 20 donors with different total embryonic numbers were selected for ELISA detection in each group. The ELISA kits of ovine ALDOB (Cat: MM-50817H2), ACAA1 (Cat: MM-50825H2), B2MG (Cat: MM-04038), ADAMTS13 (Cat: MM-05626), APO-F (Cat: MM-50834H2), and GSN (Cat: MM-0405H2) were purchased from MEIMIAN (Jiangsu, China). The assays were performed according to the manufacturer’s instructions. Briefly, 10 μL of the serum was diluted to a volume of 40 μL and incubated with 100 μL of HRP-conjugate reagent for 1 h at 37 °C. Then, the wells were washed, and 50 μL of Stop Solution was added to each well. The absorbance was read at 450 nm wavelengths using the Varioskan™ LUX Microplate Spectrophotometer (Thermo Scientific, Wilmington, DE, USA), and protein concentrations were calculated based on standard curve equations.

### Statistical analysis

The correlations between the expression levels of DEPs and total embryonic numbers were analyzed using a general linear regression model in GraphPad Prism statistical software (Version 6), with statistical significance defined as a *p*-value of < 0.05.

## Results

### Embryonic production

**Figure 1:**
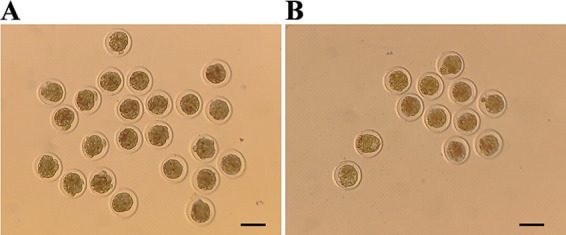
Partially collected embryos on D22 of the spontaneous estrus (SE) and D19 of the induced estrus (IE) groups. **(A)** SE group. **(B)** IE group. Scale bar: 100 μm.

Embryos from the spontaneous estrus (SE) and induced estrus (IE) groups at day 22 (D22) and D19, respectively, were collected at E6, and the majority of them were at the compacted morula stage (Figure 1). The results of the embryonic production are shown in Table 1. A total of 595 and 1,022 embryos (average: 18.6 ± 12.4 and 14.4 ± 8.7, respectively) were collected from the SE and IE groups, respectively. Among them, 432 and 681 embryos (average: 13.5 ± 8.3 and 9.6 ± 7.4, respectively) from the SE and IE groups were viable, respectively. Individuals with total embryonic yields of ≥ 20 and ≤ 13 were classified as high (HEY) and low embryonic yield (LEY) populations, respectively. In total, 9 SE and 18 IE individuals were classified as the HEY population, whereas 11 SE and 42 IE individuals were classified as the LEY population.

**Table 1:** Statistics of embryonic production after superovulation in the ewes (mean ± S.D).

Estrus mode	Spontaneous estrus	Induced estrus
Donor number	32	71
Total embryos	595	1,022
Average total embryos	18.6 ± 12.4	14.4 ± 8.7
Total viable embryos	432	681
Average viable embryos	13.5 ± 8.3	9.6 ± 7.4
High embryonic yield donor number (embryonic yield ≥20)	9	18
Average total embryos of high yield	26.5 ± 9.8	23.7 ± 7.1
Average viable embryos of high yield	19.1 ± 12.6	15.9 ± 10.3
Low embryonic yield donor number (embryonic yield ≤13)	11	42
Average total embryos of low yield	9.4 ± 3.8	8.7 ± 4.2
Average viable embryos of low yield	7.2 ± 4.7	5.3 ± 4.5

### Protein identification and quantitation

**Figure 2:**
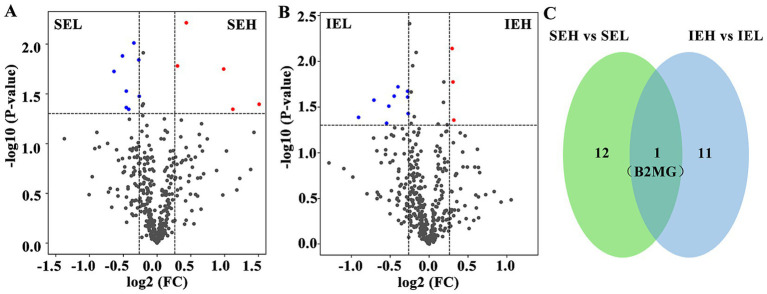
Analysis of differentially expressed proteins (DEPs) in ovine serum during superovulation. **(A)** Volcano plot showing the SEH and SEL comparison. SEH: High embryonic yield population in the SE group. SEL: Low embryonic yield population in the SE group. **(B)** Volcano plot showing the IEH and IEL comparison. IEH: High embryonic yield population in the IE group. IEL: Low embryonic yield population in the IE group. **(C)** Venn diagram showing the shared DEPs analysis results (*N* = 5).

To identify potential protein biomarkers, the serum proteome of the superovulation donor ewes was analyzed. A total of 431 proteins were identified, and 394 were annotated (Supplementary Table S1). The DEPs were screened based on the following criteria: a fold change of ≥ 1.2 or ≤ 0.83 and a *p*-value of ≤ 0.05. A total of 13 DEPs were screened in the SE group. Compared to the LEY population, five DEPs were upregulated and eight were downregulated in the HEY population of this group (Figure 2A, Table 2). In total, 12 DEPs were screened in the IE group. Compared to the LEY population, three DEPs were upregulated and nine were downregulated in the HEY population of this group (Figure 2B, Table 3). Interestingly, B2MG was the only DEP found in both the SE and IE groups (*p* < 0.05, Figure 2C).

**Table 2:** DEPs between high and low embryonic yield populations in the SE group.

Accession	Gene name	Description	Relative Abundance (SEH)	Relative Abundance (SEL)	Fold change	*P* vlaue
P52210	ALDOB	Fructose-bisphosphate aldolase B	186.48	65.44	2.85	0.0403
A0A6P3EKV9	CAT	Catalase	164.32	75.5	2.18	0.0451
A0A6P3EPA4	ACAA1	3-ketoacyl-CoA thiolase, peroxisoma	149.46	75.48	1.98	0.0178
A0A6P7E9H3	B2MG	Beta-2-microglobulin	117.32	86.92	1.35	0.0061
A0A836D582	JEQ12_014354	Uncharacterized protein	90.18	73.16	1.23	0.0166
W5PHP7	SERPIN	SERPIN domain-containing protein	93.04	111.66	0.83	0.0334
A0A6P3TN75	TMPRSS11A	Transmembrane protease serine	87.42	105.44	0.83	0.0144
B7TJ06	FN1	Fibronectin (Fragment)	87.98	111.62	0.79	0.0098
A2P2H9	VH	VH region (Fragment)	90.48	121.1	0.75	0.0453
P68214	FGA	Fibrinogen alpha chain (Fragment)	84.84	116.32	0.73	0.0298
W5PH03	FGL1	Fibrinogen like 1	86.82	119.06	0.73	0.0434
A0A6P7DTC9	ADAMTS13	Low quality protein: a disintegrin and metalloproteinase with thrombospondin motifs 13	83.48	119	0.70	0.0132
A0A836A9I6	JEQ12_013894	Uncharacterized protein	74.08	115.24	0.64	0.0189

SEH, High embryonic yield population in the SE group. SEL, Low embryonic yield population in the SE group.

**Table 3:** DEPs between high and low embryonic yield populations in the IE group.

Accession	Gene name	Description	Relative abundance (IEH)	Relative abundance (IEL)	Fold change	*P* value
A0A6P7E9H3	B2MG	Beta-2-microglobulin	108.66	87.12	1.25	0.0440
A0A6P3TLP7	APOF	Apolipoprotein F	120.58	97.50	1.24	0.0169
W5QH56	AHSG	Alpha-2-HS-glycoprotein	117.84	96.02	1.23	0.0072
A0A6P3TK65	LCP1	LOW QUALITY PROTEIN: plastin-2	85.02	102.48	0.83	0.0373
W5NT24	TNXB	Tenascin XB	89.24	108.10	0.83	0.0213
A0A836CWY9	JEQ12_003532	Uncharacterized protein	88.18	106.84	0.83	0.0246
A0A836CX49	JEQ12_003710	Uncharacterized protein	92.46	122.12	0.76	0.0190
W5PXX3	F13B	Coagulation factor XIII B chain	81.00	110.56	0.73	0.0240
W5Q9T3	LMNB1	Lamin B1	84.28	120.68	0.70	0.0310
W5PEE9	LAMP1	Lysosomal associated membrane protein 1	89.86	131.32	0.68	0.0476
F2YQ13	GSN	Gelsolin OS=*Ovis aries*	74.30	121.60	0.61	0.0266
A0A835ZRY0	JEQ12_007502	Ig-like domain-containing protein	70.10	131.54	0.53	0.0411

IEH, High embryonic yield population in the IE group. IEL, Low embryonic yield population in the IE group.

**Figure 3:**
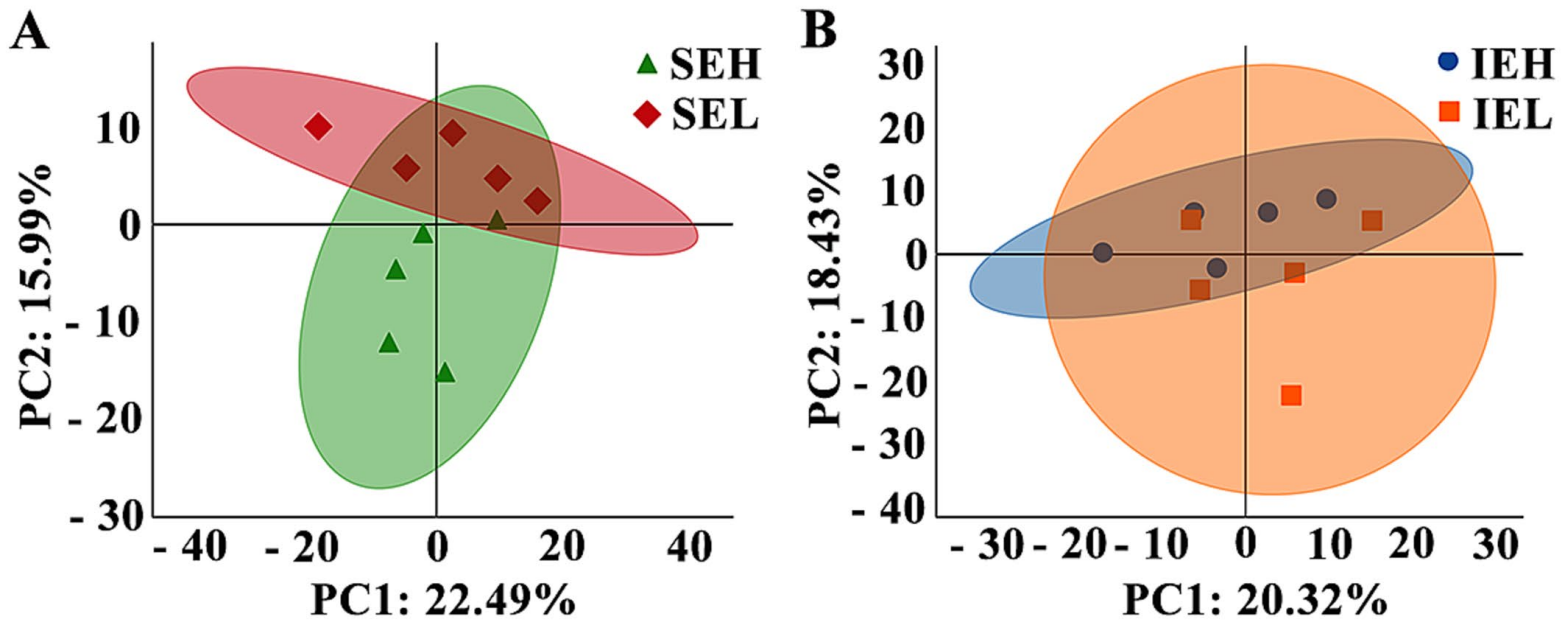
Principal component analysis (PCA) of ovine serum proteome during superovulation. **(A)** The SEH and SEL comparison. SEH: High embryonic yield population in the SE group. SEL: Low embryonic yield population in the SE group. **(B)** The IEH and IEL comparison. IEH: High embryonic yield population in the IE group. IEL: Low embryonic yield population in the IE group. The colored points in the graph indicate the individual samples (*N* = 5).

To analyze the relationship between the individuals, a principal component analysis (PCA) was performed. As shown in Figure 3, principal component 1 (PC1) and principal component 2 (PC2) accounted for 22.49 and 15.99% of the variation in the SE group comparison (Figure 3A) and 20.32 and 18.43%, respectively, of the variation in the IE group comparison (Figure 3B).

### Functional analysis of DEPs

First, Gene Ontology (GO) annotation was used for the functional annotation of the DEPs. For the upregulated DEPs from the HEY population in the SE group, 17 terms were enriched, of which 13 were significantly enriched, including antigen processing and presentation of peptide antigen via MHC class I, glycolytic process, and positive regulation of cell division (Supplementary Table S2). For the downregulated DEPs, 12 terms were enriched, of which 9 were significantly enriched, including extracellular region, blood coagulation, and extracellular matrix organization (Supplementary Table S3).

For the upregulated DEPs from the HEY population in the IE group, nine terms were enriched, of which six were significantly enriched, including antigen processing and presentation of peptide antigen via MHC class I, MHC class I protein complex, cysteine-type endopeptidase inhibitor activity (Supplementary Table S4). For the downregulated DEPs, 49 terms were enriched, of which 43 were significantly enriched, including regulation of JUN kinase activity, blood coagulation, fibrin clot formation, and extracellular matrix organization (Supplementary Table S5).

Afterward, Kyoto Encyclopedia of Genes and Genomes (KEGG) enrichment analysis was performed to enrich the functional pathways of the DEPs. For the upregulated DEPs from the HEY population in the SE group, 24 pathways were enriched, of which 11 were significantly enriched, including peroxisome, biosynthesis of unsaturated fatty acids, valine, leucine, and isoleucine degradation (Supplementary Table S6). For the downregulated DEPs, 12 pathways were enriched, including ECM-receptor interaction, focal adhesion, and the PI3K-Akt signaling pathway (Supplementary Table S7). For the upregulated DEPs from the HEY population in the IE group, six pathways were enriched, including antigen processing and presentation, Epstein–Barr virus infections (Supplementary Table S8). For the downregulated DEPs, 15 pathways were enriched, including Fc gamma R-mediated phagocytosis, ECM-receptor interaction, and the PI3K-Akt signaling pathway (Supplementary Table S9).

### Protein–protein interaction (PPI) network analysis of DEPs

**Figure 4:**
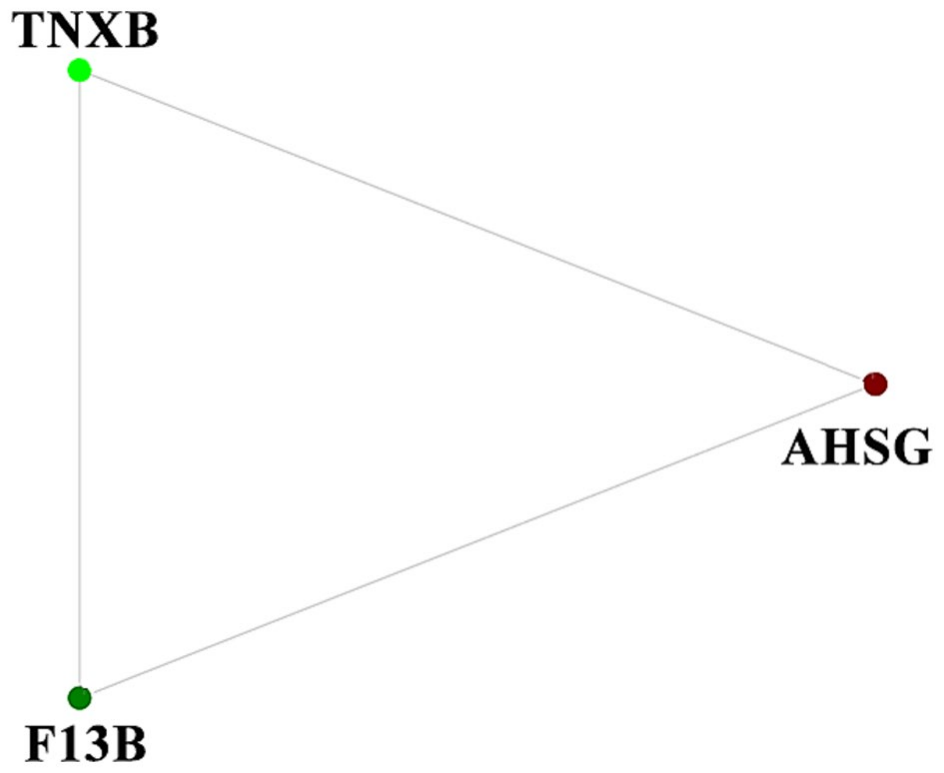
The protein–protein interaction (PPI) network analysis of the DEPs in the IE group.

To discover the blood protein interaction network during ovine superovulation, a protein interaction network was constructed using the DEPs. Three DEPs in the IE group, namely TNXB, AHSG, and F13B, were found to interact, each of which was involved in different pathways (Figure 4).

### Validation of partial DEP expression levels using the ELISA

**Figure 5:**
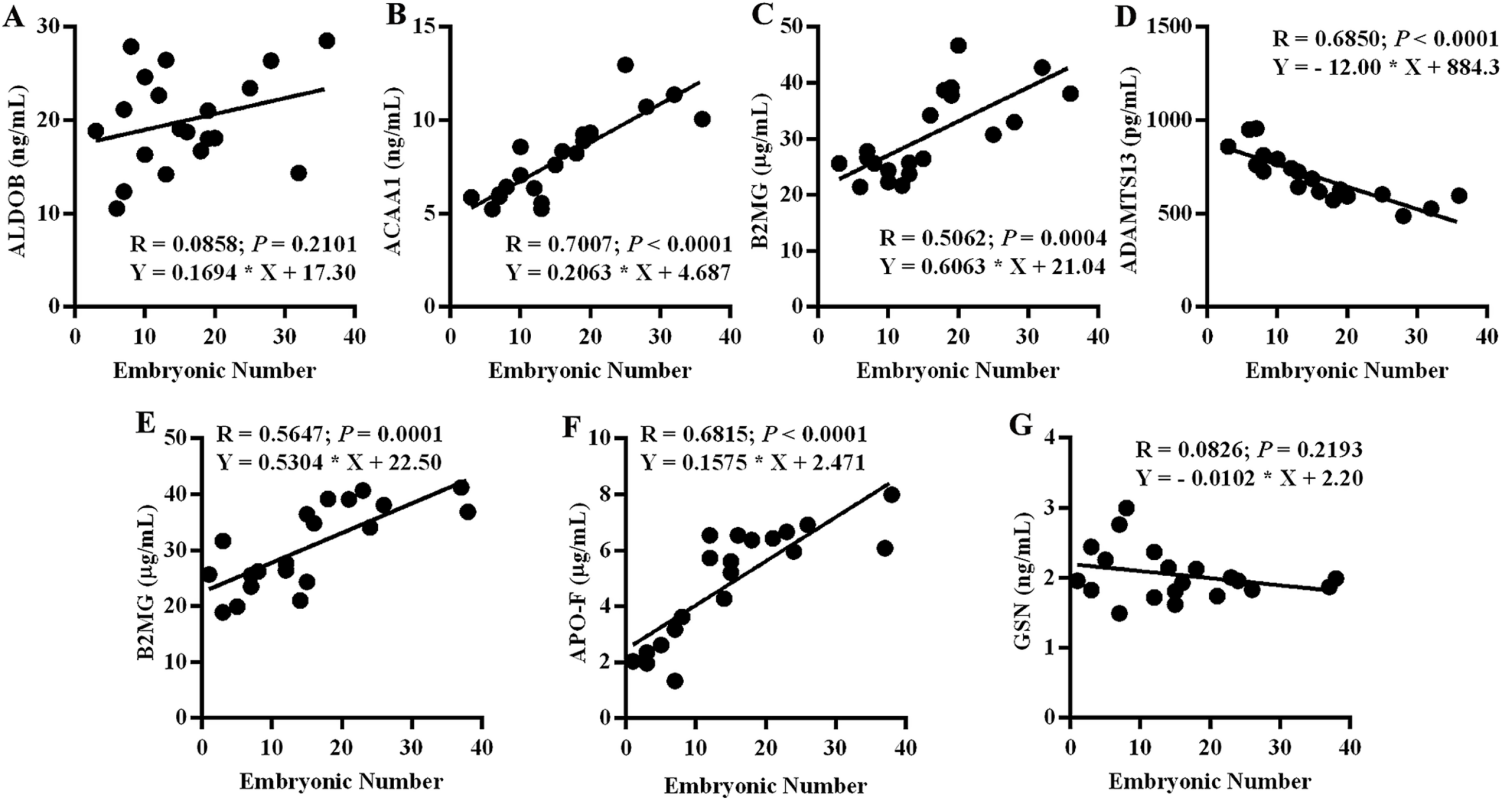
ELISA results for the partial DEPs. The correlation between DEP expression levels and embryo yield was analyzed using linear regression. **(A)** ALDOB in the SE group. **(B)** ACAA1 in the SE group. **(C)** B2MG in the SE group. **(D)** ADATMS13 in the SE group. **(E)** B2MG in the IE group. **(F)** APO-F in the IE group. **(G)** GSN in the IE group. SE, Spontaneous Estrus; IE, Induced Estrus (*N* = 19).

To confirm the validity and reliability of the proteome and identify the potential biomarkers for ovine superovulation, ELISA was utilized to assess the expression levels of partial DEPs. We assessed the expression levels of ALDOB, ACAA1, B2MG, and ADAMTS13 in the SE group and B2MG, APO-F, and GSN in the IE group. Subsequently, linear regression analyses were conducted using the total embryonic number. As shown in Figure 5, ACAA1, B2MG, and ADAMTS13 in the SE group and B2MG and APO-F in the IE group exhibited significant linear correlations with the total embryonic number (*p* < 0.001), whereas ALDOB in the SE group and GSN in the IE group exhibited only mild linear correlations (*p* > 0.05). ACAA1 and B2MG in the SE group and B2MG and APO-F in the IE group were positively correlated with the total embryonic number, while ADAMTS13 in the SE group was negatively correlated with the total embryonic number (Figure 5).

## Discussion

In this study, the relationship between blood proteins and the embryonic production outcome of ovine superovulation was examined. Our results showed that the DEPs between HEY and LEY populations are involved in the metabolism of unsaturated fatty acids and amino acids, oxidative stress, ECM–receptor interaction, and the PI3K-Akt signaling pathway. Some of these pathways have been demonstrated to affect the development of embryos and/or oocytes. Partial DEPs, such as ACAA1, B2MG, APO-F, and ADAMTS13, were identified as potential biomarkers for ovine superovulation.

During ovine superovulation, embryos can be collected from either the oviducts or the uterus. In general, E3 embryos are collected from the fallopian tubes. In addition, E6 ~ 6.5 embryos are collected from the uterus. Embryo collection from the uterus has less reproductive impact on donors than collection from the oviducts. In this study, we collected embryos at E6 from both groups. The majority of them were in the compacted morula stage. However, the average total and viable embryos collected from the SE group were higher than those collected from the IE group. This finding might be attributed to the mode of estrus synchronization, which was induced by P4. Excessive P4 would inhibit the development of follicles. In a monkey model, P4 implantation in the ovaries was found to inhibit follicular development and suppress granulosa cell mitosis and estrogen secretion (E2) (12). E2, in turn, is essential for the development of oocytes.

Fatty acids are one of the energy sources for the development of oocytes and embryos. Previous research on goat superovulation revealed that supplementation with unsaturated fatty acids is a feasible way to prevent premature luteal regression and improve embryo quality (13). In this study, we discovered that fatty acid metabolism-associated ACAA1 exhibits distinct expression levels between HEY and LEY populations. ACAA1 is a key regulator of fatty acid *β*-oxidation in peroxisomes, a process responsible for the elongation and degradation of fatty acids (14). In breast cancer, ACAA1 inhibition has been shown to restrain the proliferation of cancer cells (15). A higher level of ACAA1 in ewes might be an indicator of improved fatty acid metabolism, which could further enhance embryonic development.

Carbon sources such as fructose and glucose are important for the development of embryos and oocytes. In *in vitro* maturation research on porcine oocytes, supplementation with glucose and fructose has been shown to significantly promote various key processes, such as germinal vesicle breakdown, maturation to metaphase II, penetration by spermatozoa, and male pronuclear formation (16). The addition of 3 mmol/L glucose to *in vitro* fertilized embryos significantly enhances their developmental competence (17). Mechanistically, fructose is transported into hepatocytes and phosphorylated to F1P, which is then cleaved by ALDOB into dihydroxyacetone phosphate and glyceraldehyde (18). Our study found that ALDOB was upregulated in the HEY population of the SE group, suggesting that a higher fructose metabolic capacity might be beneficial for the development of oocytes and embryos.

Oxidative stress is a crucial factor impacting the ovum and embryonic development. In the SE group, the expression of CAT was upregulated in the HEY population. Some studies have shown that higher CAT levels are associated with improved developmental efficiency of oocytes and embryos (19–21). We surmised that assessing blood oxidative stress indices could effectively aid in donor selection during superovulation.

Interestingly, we found that B2MG was upregulated in the HEY populations of both groups. Along with major histocompatibility complex class I, B2MG plays a central role in the biological processes of mammals. For instance, a B2MG knockout mouse exhibited impaired reproductive performance (22), whereas a B2MG knockout pig died within 4 weeks due to unexpected disease processes (23). In infertile patients, B2MG concentration in the follicular fluid has been associated with the assisted reproductive technology outcomes, with higher levels in the positive group (24). In women with polycystic ovary syndrome, higher urine B2MG levels have been associated with gestational diabetes mellitus during mid-pregnancy (25). However, the underlying mechanism requires further investigation.

## Conclusion

In conclusion, we showed that some blood proteins are associated with ovine superovulation outcomes. The DEPs identified were associated with the metabolism of fatty acids, saccharides, amino acids, oxidative stress, and antiviral pathways. Furthermore, ACAA1, B2MG, and ADAMTS13 in the SE group and B2MG and APO-F in the IE group could be used as potential biomarkers for donor selection during ovine superovulation. In the future, our findings need to be validated in large samples from pastures.

## Data Availability

The datasets presented in this study can be found in online repositories. The names of the repository/repositories and accession number(s) can be found in the article/Supplementary material.

## Footnotes

^1^ https://arriveguidelines.org

## Supplementary material

Supplementary Table S1 Expressions of identified proteins in ovine serum by proteome analysis at superovulation.

Supplementary Table S2 GO result of up-regulated differentially expressed proteins at SEH vs SEL comparation.

Supplementary Table S3 GO result of down-regulated differentially expressed proteins at SEH vs SEL comparation.

Supplementary Table S4 GO result of up-regulated differentially expressed proteins at IEH vs IEL comparation.

Supplementary Table S5 GO result of down-regulated differentially expressed proteins at IEH vs IEL comparation.

Supplementary Table S6 KEGG result of up-regulated differentially expressed proteins at SEH vs SEL comparation.

Supplementary Table S7 KEGG result of down-regulated differentially expressed proteins at SEH vs SEL comparation.

Supplementary Table S8 KEGG result of up-regulated differentially expressed proteins at IEH vs IEL comparation.

Supplementary Table S9 KEGG result of down-regulated differentially expressed proteins at IEH vs IEL comparation.
